# Analysis of the Relationship between the Referral and Evolution of Patients with Type 2 Diabetes Mellitus

**DOI:** 10.3390/ijerph15071534

**Published:** 2018-07-20

**Authors:** Camilo Alvarez, Cecilia Saint-Pierre, Valeria Herskovic, Marcos Sepúlveda

**Affiliations:** Department of Computer Science, Pontificia Universidad Católica de Chile, Santiago 7820436, Chile; cealvarez@uc.cl (C.A.); csaintpierre@uc.cl (C.S.-P.); vherskov@ing.puc.cl (V.H.)

**Keywords:** adherence, Type 2 Diabetes Mellitus, healthcare, organizational research, referrals

## Abstract

Type 2 Diabetes Mellitus (T2DM) is a chronic disease that has risen in prominence in recent years and can cause serious complications. Several studies show that the level of adherence to different types of treatment has a direct correlation with the positive evolution of chronic diseases. While such studies relate to patient adherence to medication, those that concern adherence to medical appointments do not distinguish between the different disciplines that attend to or refer patients. This study analyses the relationship between adherence to referrals made by three distinct disciplines (doctors, nurses, and nutritionists) and the results of HbA1c tests from a sample of 2290 patients with T2DM. The aim is to determine whether a relationship exists between patient improvement and the frequency with which they attend scheduled appointments in a timely manner, having been previously referred from or to a particular discipline. Results showed that patients tended to be more adherent when their next appointment is with a doctor, and less adherent when it is with a nurse or nutritionist. Furthermore, patients that remained stable had higher rates of adherence, whereas those with lower adherence tended to be more decompensated. The results can enable healthcare professionals to monitor patients and place particular emphasis on those who do not attend their scheduled appointments in a timely manner.

## 1. Introduction

Type 2 Diabetes Mellitus (T2DM) is a chronic disease characterized by the inability of the pancreas to produce sufficient amounts of insulin, or when the organism does not use the insulin it produces in an efficient manner. Globally, T2DM affects more than 400 million people. In Chile, approximately 11% of the population has been diagnosed with this disease, while in Latin America, the number of people with diabetes is expected to increase 65% by 2040 [1]. Only about 36% of diabetics in Chile have their glucose levels under control, i.e., achieve a glycated haemoglobin (HbA1c) level under 7% [2].

Healthcare experts have created protocols to establish care guidelines for patients, as well as the provision of advice and procedures for healthcare professionals to follow, in order to stabilize patients and thereby prevent related complications from arising. In Chile, there is a government guide that outlines a multidisciplinary approach, including regular check-ups from a team of professionals composed of at least one doctor, one nurse, and one nutritionist [2].

The World Health Organization defines adherence to treatment as the extent to which the behavior of a person, in relation to their diet, consumption of medication, and general lifestyle, corresponds to recommendations provided by healthcare professionals [3]. Healthcare professionals are able to intervene in the habits and self-care of patients by means of medical appointments [4]. It has been demonstrated that education in diabetes and patient self-management give rise to improved long-term results in relation to diabetes [5,6,7]. Attending appointments in a timely manner helps to generate a link between patient and healthcare teams. This link strengthens positive communication between the two parties and is particularly important in terms of ensuring effective treatment [3]. In general, diabetics display low levels of adherence to taking medicine, as well as to attending appointments in a timely manner [8,9]. The main reasons that affect whether patients attend appointments include ignorance about the disease, differing specialist opinions, constant consumption of medication, dissatisfaction with the healthcare attention provided, a lack of medical resources and services available, socioeconomic and educational levels, and fear of insulin injections [10,11].

The literature shows a correlation between adherence to medication and the positive evolution of patients, e.g., using statin treatment to reduce low-density lipoprotein (LDL) cholesterol [12], or in the reduction of cholesterol and adherence to treatment using cholestyramine [13]. Studies have also been conducted into the relationship between timely attendance to appointments and medical results, e.g., the increase in the rate of heart attacks and its correlation with low rates of adherence to appointments in a timely manner [14].

Regarding T2DM, the majority of articles pertain to adherence to medication. Patients tend to follow professional indications when it comes to taking just one pill [15], and when using only one type of drug [16]. Studies that concern adherence to medication provide evidence that compliance with professional indications produces better results. However, visits to healthcare professionals are also an important factor in the positive evolution of patients. Therefore, patients should not only adhere to professional advice related to the consumption of medication, but also to appointments [5]. In terms of adherence to the latter, it is important that a relationship is established between doctor and patient. Among patients who tend to miss their appointments, those who experience more serious effects of T2DM usually classify the quality of their visit to the doctor as insufficient, whereas patients who are more stable classify their appointments as positive [17]. Furthermore, adherence to medication and to HbA1c tests is lower among patients who fail to attend their appointments [17]. An increase in the rate of non-attendance of appointments raises the probability that patients become decompensated [18]. However, these studies make no distinction between the different professionals who attend to patients and, rather, focus on medication and ignore the particular medical discipline in question.

A broad range of methods have been used to measure patient adherence. One of these focuses on asking professionals and patients about the degree of patient adherence to treatment. However, both professionals and patients tend to overestimate adherence [19,20,21]. Another method is to conduct standardized questionnaires [22], which help to identify characteristics of patients but do not act as good predictors of adherence [23]. An additional metric calculates the proportion of appointments attended in a timely manner in relation to the total number of appointments made for a particular patient. To correctly calculate this metric, it is important to differentiate between appointments that are cancelled, rescheduled, or which the patient was unable to attend due to unforeseen circumstances. Moreover, it is important to establish a margin of time to determine whether the patient attended the appointment “on time”. This metric can be used to analyze the different professional disciplines that treat patients. Furthermore, it is possible to analyze adherence to the level of referrals or transitions of patients among the distinct disciplines. The adherence metrics studied only analyze appointment attendance, regardless of which professional the appointment was with or which discipline made the patient referral.

A number of different methods have been studied to increase adherence, such as the use of reminder systems to help patients to remember to take their medication and attend their appointments [24]. In Chile, one study showed that a telephone support model increases treatment adherence, providing evidence that this rise in compliance increases the probability that patients are stabilized, as well as reducing the number of emergency visits to primary healthcare centers [25].

This paper aims to study whether adherence to appointments is related to the evolution of patients with diabetes. We analyze compliance to medical referrals, which occur when professionals refer patients to either the same or a different discipline. To undertake the study, authors used patient records that were obtained from the information systems of three family healthcare centers belonging to the Áncora UC network, which is part of the Pontificia Universidad Católica de Chile.

## 2. Materials and Methods

### 2.1. Context

The Chilean healthcare system consists of public and private healthcare providers. In the public sector, primary healthcare is the foundation of the system and its primary healthcare centers provide a range of promotional, preventative, treatment- and rehabilitation-based services.

Family healthcare centers (CESFAMs) are based on a model that focuses on family and community health. One of the main tasks of CESFAMs is to treat patients with chronic diseases that require ongoing treatment (e.g., T2DM). The main challenge facing CESFAMs is a shortage of professionals to cover the required healthcare demand.

The Universal Access Plan to Explicit Health Guarantees, also known as the AUGE or GES program, established by the Ministry of Health in 2005, guarantees free access and attention to people experiencing any of 80 health-related problems, including T2DM. Patients with diabetes who are treated in the public system are treated under the same protocols, as defined by the Ministry of Health, as well as having access to the same medicine.

To receive healthcare attention in a CESFAM, a patient must simply visit their local center and make an appointment. As per CESFAM rules, patients cannot make an appointment more than a month in advance, due to the fact that patients frequently fail to attend appointments that are made with such lengthy anticipation. In the case of diabetic patients, at the end of a check-up appointment, the professional tells him/her when to schedule the next appointment. The professional can schedule the appointment directly in the information system. Meanwhile, patients can only schedule the appointment through the service desk of the center. In some cases, patients must have exams before scheduling the next check-up. Furthermore, in some cases, the system will not allow the patient to book an appointment too far in advance. In those cases, patients must attend the healthcare center to book an appointment.

### 2.2. Data Collection

Data was collected from patients diagnosed with T2DM from across three CESFAMs pertaining to the university health network. This data included demographic information related to the patients, in addition to their appointments and tests. Only patients with T2DM who had their cardiovascular periodic appointments (CVPA) or check-ups were considered. These appointments or check-ups were carried out by any of the three professionals relevant to this study: doctor, nurse, or nutritionist [26]. Each time one of the three aforementioned professionals completed a CVPA, they must indicate which professional the patient should visit during his/her next check-up, as well as an approximate date of that appointment. The transitions of a patient between the distinct disciplines for separate appointments could be described as medical referrals, as long as the professional who they are scheduled to see during the next check-up is clearly indicated within this process. This study analyzed adherence to these medical referrals. The data collected pertained to the period January 2012 to November 2016.

### 2.3. Procedures

We carried out an observational, descriptive, and analytical study to analyze the relationship between the adherence to medical referrals made by distinct disciplines and the results of a patient evolution metric. The aim was to determine whether a relationship existed between patient improvement and the frequency with which they attended scheduled appointments in a timely manner, having been previously referred from or to a particular discipline.

To achieve this goal, we selected primary care patients from three family healthcare centers in Santiago, Chile, under treatment for T2DM and considered their cardiovascular periodic appointments (CVPA). To analyze the data, we proposed a method in which adherence to referrals made by three distinct disciplines (doctors, nurses, and nutritionists) was measured as the proportion of CVPAs attended in a timely manner with the indicated professional in relation to the total number of CVPAs. The HbA1c test results were used as patient evolution metric. Four evolution segments of patients with T2DM, according to their respective evolution over time, were considered. Proportion tests were conducted to compare the adherence of each segment with that of the total population.

#### 2.3.1. Log Creation

An event log can be seen as a collection of cases that contain information about all the activities undertaken for a particular process [27]. In this study, each case grouped all CVPAs completed in relation to a particular patient. Each CVPA was defined according to the following fields:

- Unique patient identification
- Date and time of appointment
- Reason for healthcare visit
- Name of professional attending to patient
- Discipline or role of professional
- Month of next patient check-up
- Role of professional of next patient check-up

To create an event log, data was extracted from the information systems used by healthcare professionals to record information that related to completed patient CVPAs. This information considered, among other aspects, the aforementioned fields for each CVPA. Data cleansing was conducted in conjunction with a patient filter, taking into account only those cases whose appointment records contained all the information from the aforementioned fields.

#### 2.3.2. Patient Evolution Metric

The HbA1c test was used to control the level of glucose in the blood of a patient with diabetes. Ministry of Health protocols stipulate that patients are stable when HbA1c levels are lower than 7% [2]. In addition, these protocols establish two levels of decompensation: first, moderate decompensation, when test values are between 7% and 9%, inclusive; and second, high decompensation, when test values exceed 9% [2].

In addition to the appointment records, data was collected in relation to measurements from the HbA1c test. These tests were carried out regularly, with periodicity depending on the severity of the patients. The segmentation described in Reference [26] was applied using the HbA1c test result history of each patient to create four evolution segments of patients with T2DM, according to their respective evolution over time. These groups included: *stable* patients (called compensated patients in Reference [26]), whose measurement average was less than 7% and at the most had one measurement between 7% and 9%; *improved* patients, who initially had an HbA1c of over 7%, but who reduced their value to below 7% during the course of treatment; *moderately decompensated* patients, who had no measurements that exceeded 9% (but were neither stable nor improved); and, *highly decompensated* patients, who had a measurement over 9% (but showed no improvement).

#### 2.3.3. Medical Referral Adherence Metric

The information systems of CESFAMs save a record of each patient appointment, including the month in which he/she must return for a CVPA, and the role of the professional with whom he/she should have their next check-up. This system enables patients to be referred to other disciplines, or a professional of the same discipline. Adherence to referrals was measured as the proportion of CVPAs attended in a timely manner with the indicated professional in relation to the total number of CVPAs [28]. A margin of time window of ±4 months [26] was considered, beginning from the last day of the month in which a new check-up was made for the patient. A patient is considered to have adhered to the referral if he/she attended the next CVPA with the indicated professional and in accordance with the stipulated time, taking into account the margin of time window. For the purpose of this study, we considered it only necessary for the patient to attend their CVPA with the relevant discipline (doctor, nurse, or nutritionist) rather than with the same individual professional who conducted the previous check-up. All CVPAs carried out during the period of scope of this study, and for which the subsequent patient check-up took place within the same period (taking into account the margin of time window), were considered. Within the considered period, the first CVPA of each patient was discarded because it is not possible to know for certain if the patient attended the first check-up that appeared in the appointment records in a timely manner. Similarly, all CVPAs with the next check-up scheduled for a time that fell outside the period of scope of this study were discarded.

$A_{de}$ represented the number of adherent check-ups in which discipline d referred the patient to discipline e; and $R_{de}$ represented the number of referrals that discipline d made to discipline e, with d,e∈{Doctor, Nurse, Nutritionist}, i.e., the total number of adherent check-ups in relation to the total number of referrals. 

General adherence C was, then, defined as follows:

(1) $$\;C\;=\;\frac{\sum_{d}\sum_{e}A_{de}}{\sum_{d}\sum_{e}R_{de}}$$

This adherence was compared with the grouped and specific referrals in order to analyze pertinent trends. Adherence to referrals to which a patient was subjected was divided into two categories:

- Grouped referrals: This category was separated into two subcategories: (1) adherence to referrals from a specific discipline, regardless of which professional is conducting the next check-up. Adherence $F_{d}$ from a discipline d was calculated as follows:
  (2) $$\;F_{d}\;=\;\frac{\sum_{e}A_{de}}{\sum_{e}R_{de}}\;$$
  The second subcategory was: (2) adherence to a specific discipline, regardless of which discipline referred the patient. Adherence $T_{e}\;$to a discipline e was calculated as follows:
  (3) $$\;T_{e}\;=\;\frac{\sum_{e}A_{de}}{\sum_{e}R_{de}}\;$$
  The aim was to analyze whether patients were more adherent to referrals made by a particular discipline, or in attending referrals undertaken to a particular discipline.
- Specific referrals: Patient compliance with specific referrals was measured when one particular discipline made a referral specifically to a given discipline (either the same discipline or another discipline). Adherence to referral $S_{de}$ from discipline d to discipline e was defined as:
  (4) $$\;S_{de}\;=\;\frac{A_{de}}{R_{de}}\;$$

For the three disciplines considered in this study, a set of six metrics was obtained for grouped referrals, and nine metrics for specific referrals.

#### 2.3.4. Patient Selection

The information systems of the three CESFAMs studied contained records of 3369 patients diagnosed with T2DM. The application of the first filter helped to identify the patients who had taken at least two HbA1c tests. The aim of the filter was to measure the evolution of these patients and classify them according to the aforementioned segments. Subsequently, a second filter was applied according to the number of CVPAs completed by the patients, recording all who had conducted at least two check-ups during the period of study. This was due to the fact that, to be able to say that a patient’s check-up was completed in a timely manner, the previous appointment record was necessary in order to know the date of the patient’s next check-up. Having applied both filters, a total of 2290 patients was obtained, in conjunction with a total of 23,761 referrals. Patients were divided equally between the three centers. The age range of the sample was from 20 to 95 (average age is 62.9). However, the sample consisted primarily of adults between 50 and 80 (72%), out of which 58.5% of the sample were women. On average, the patients had T2DM for 6.9 years. While 475 patients presented neither severe conditions nor comorbidities, 1086 of them presented at least one condition of higher severity, and 1044 presented at least one comorbidity. For example, around 1600 underwent diabetic foot care and 550 suffered from overweight or obesity

#### 2.3.5. Adherence Comparison

A proportions test was conducted to compare the adherence percentages of each segment with that of the total population. In addition, the adherence percentage of each segment was compared, using the same test, in order to identify the statistical differences among the patients from different segments, utilizing both the grouped and specific referral adherence metrics.

#### 2.3.6. Patient Data Analysis

A questionnaire was carried out on 31 patients who attended one of the three CESFAMs covered by this study. This questionnaire consisted of open questions about appointment attendance, reasons for attending the healthcare center, the process of making appointments, previous test results, and the most recent test result. To analyze the answers, a thematic analysis was applied to the responses [29]. The aim of the questionnaire was to complement the results and understand the motives behind patient adherence and non-adherence to appointments.

### 2.4. Ethical Considerations

The study protocol was approved by the university ethics committee (13-467 and 160928001). Data from information systems were anonymized, interview participants were explicitly informed about the aim of the research, and no personal information was requested.

## 3. Results

### 3.1. Overall Adherence

Overall adherence among the sample population was 63.97%, taking into account the total of 23,761 referrals. The general adherence was broken down at three levels: first at the level of grouped referrals, then at the level of specific referrals, and finally at the level of evolution segments. Subsequently, the relationship between referrals and evolution segments was analyzed.

### 3.2. Grouped Referral Adherence

Table 1 and Table 2 show the level of adherence to group referrals: from one discipline to any other discipline, and from any discipline to a specific one, respectively. In referrals from one discipline, the trend was that referrals from doctors showed lower adherence than general overall adherence (*p*-value = 0.0037), while referrals from nutritionists showed greater adherence (*p*-value < 0.0001) compared to the general overall adherence. On the other hand, referrals to doctors showed greater adherence compared to the general overall adherence, and referrals to other disciplines showed lower adherence than general overall adherence. All of these differences in adherence were statistically significant (*p*-value < 0.0001).

### 3.3. Specific Referral Adherence 

Table 3 shows adherence to specific referrals. Of the referrals made by doctors, adherence to the Doctor–Nurse and Doctor–Nutritionist referrals was lower than overall adherence, which itself was consistent with adherence to grouped referrals $F_{\mathrm{doctor}}$. However, adherence to the doctor–doctor referral was greater than overall adherence. the specific nutritionist–nutritionist referral had the lowest adherence out of all specific referrals. conversely, adherence to the nutritionist–doctor referral was greater than adherence of the overall population. Regarding specific referrals to a given discipline, all referrals made to doctors ($S_{\mathrm{doctor},\mathrm{doctor}}$, $S_{\mathrm{nurse},\mathrm{doctor}}$, $S_{\mathrm{nutritionist},\mathrm{doctor}}$) showed greater adherence than the overall adherence, and referrals to nurses ($S_{\mathrm{doctor},\mathrm{nurse}}$, $S_{\mathrm{nurse},\mathrm{nurse}}$) or nutritionists ($S_{\mathrm{doctor},\mathrm{nutritionist}}$, $S_{\mathrm{nutritionist},\mathrm{nutritionist}}$) showed lower adherence, with the exception of the nurse–nutritionist ($S_{\mathrm{nurse},\mathrm{nutritionist}}$) and nutritionist–nurse ($S_{\mathrm{nutritionist},\mathrm{nurse}}$) referrals, for which adherence is similar to the overall rate. Even though the majority of the specific referrals to one particular discipline matched the trend related to their grouped referral $T_{d}$, only certain specific referrals from one particular discipline matched the trend of their grouped referral $F_{d}$, while other referrals showed adherence that differed from that indicated in their respective trends.

### 3.4. Specific Referral Adherence and Evolution

Table 4 shows the level of adherence considering the grouped referrals from a discipline, $F_{d}$, and the evolution segments. In addition, it shows the general adherence of each segment. Highly decompensated patients showed the lowest adherence, with adherence levels lower than the overall population in all types of referrals. Moderately decompensated and stable patients showed a similar adherence to the general rate of adherence, with the exception of doctor–nurse (*p*-value = 0.0055) and nutritionist–nurse (*p*-value = 0.0023) referrals, in which the adherence of stable patients was greater than the adherence of the overall population with regard to these referrals. improved patients showed greater adherence than the overall population.

Adherence to grouped referrals followed the same pattern as the overall adherence of the segments, with the exception of the nutritionist–any referral, in which the stable and moderately decompensated patients showed greater adherence than improved patients. in this referral, moderately decompensated (*p*-value = 0.0252) and stable (*p*-value < 0.0001) patients showed greater adherence than the general adherence of the respective segment. when nutritionists made referrals, stable and moderately decompensated patients were the most compliant. 

Alternatively, stable patients showed lower adherence in the doctor–any referral with regard to the general adherence of this segment (*p*-value = 0.0452). among the referrals made from doctors, the doctor–doctor referral showed greater adherence than the general adherence of all their respective groups, while the doctor–nurse and doctor–nutritionist referrals show lower adherence that the general rate in each group, which was consistent with that described in Section 3.3.

Table 5 shows the level of adherence considering the grouped referrals to a discipline, $T_{d}$, and the evolution segments. Once more, in the any–nutritionist referral, stable patients showed greater adherence than improved patients, which differed from the general adherence trend of each segment. in particular, the nurse–nutritionist referral showed greater adherence among stable patients than improved patients. referrals to nurses showed lower adherence than the general adherence in each group, with the exception of the nutritionist–nurse referral, in which stable patients showed greater adherence than their respective general adherence (*p*-value = 0.0079). Generally, patients tended to show greater adherence when their next check-up was with a doctor, and lower adherence when they were scheduled to visit a nurse or nutritionist. This trend was repeated across all evolution segments.

### 3.5. Comparison of Adherence among Evolution Segments

By comparing adherence among the evolution segments, a difference could be seen between highly decompensated patients, in almost all specific and grouped referrals, and the groups with greatest adherence, i.e., between stable and improved patients. in the nutritionist–nurse referral, stable patients showed greater adherence than the rest of the segments, thereby differing significantly, even from improved patients. Conversely, in the doctor–nurse referral, highly decompensated patients showed adherence that was statistically lower than all other segments, differing even from moderately decompensated patients.

### 3.6. Patient Data Analysis

To complement the results, the responses from the questionnaire were analyzed. Regarding the scheduling of check-ups, patients that failed to make an appointment when leaving the healthcare center claimed that this was due to the lack of tests, that the professional made the appointment for them, that the system would not allow it (due to a problem or because there was a specific period in which to make an appointment), or because the patient made the appointment according to his/her own preference (when they needed the check-up), i.e., without a specific date being provided by the professional. The majority of the questionnaire respondents claimed they did not make appointments due to problems with the system. Regarding failure to attend appointments, respondents claimed that they did not attend due to unforeseen circumstances, a lack of tests, and complications related to other illnesses.

Patients were asked for the results of their last HbA1c test, as well as for their respective evolution in relation to previous tests. By cross referencing the responses with the results of the last test and their evolution compared to previous tests, it could be seen that the majority of respondents had positive results in their last test and were experiencing an improvement over time. Another of the most common groups of responses was that of patients who, in their last test, had deteriorated, and had been doing so progressively; and patients who, in their last test, were well and had maintained a stable level of health.

Regarding preferences for a particular discipline, the majority of respondents claimed that they had no preference regarding the three professions, since each one has a distinct outlook. Other respondents claimed to prefer seeing a doctor, since they were able to prescribe medication or treat other illness beyond just diabetes. It was also possible to observe from the responses that there was a preference for appointments with a nurse and an aversion to those with a nutritionist. According to respondents, this aversion was because patients did not view the attention they received from nutritionists in a favorable light, they only go when they had put on weight, and they did not take into account the professional’s recommendations.

## 4. Discussion

In contrast to the research outlined in References [14,17,18], this study has analyzed adherence with particular emphasis on referrals between distinct healthcare professionals. Accordingly, it was possible to identify the preference of professionals who attended to patients. In general, patients showed lower adherence when they were referred by doctors. In contrast, if patients were referred to a doctor, they tended to be more adherent than if they were referred to any other discipline. This concurred with the responses of some of the patients who completed questionnaires with regard to their preference for attending appointments with a doctor and their aversion to seeing a nutritionist.

The level of adherence to referrals made by doctors was lower than the general adherence of the overall population. However, the doctor–doctor referral showed an adherence level greater than that of the general adherence. This could be explained by the fact that there were more appointments available with doctors than for other disciplines, which meant that check-ups with these professionals were the most common type of appointment in the healthcare centers studied. Furthermore, according to responses provided by some patients, there was a preference for appointments with doctors because some patients have other illnesses in addition to diabetes, which could be treated by these professionals. Moreover, the doctor was the one professional who could prescribe medication, which was one of the reasons why patients made appointments with doctors more frequently than with other disciplines. 

Conversely, the level of adherence to referrals made by nutritionists was lower than that of the general adherence of the population, with the exception of the nutritionist-doctor referral. Similarly, referrals made to nutritionists were fewer. There was only limited availability for appointments with a nutritionist in the CESFAMs included in this study, which was why referrals that involve a nutritionist were less common compared to other referrals. This was possibly due to the difficulty of making an appointment with this type of professional. In addition, certain questionnaire respondents did not view the attention provided by nutritionists as favorable, which resulted in fewer patients attending their appointments. According to the findings of Reference [17], unfavorable views of attention provided by healthcare professionals was related to lower levels of adherence. 

Regarding the evolution segments, stable patients showed greater adherence than all other segments, including improved patients, in referrals that involve nutritionists. To treat diabetes, it was important that patients led a healthy life that includes a balanced diet, exercise, and the consumption of medication [30]. In such cases, nutritionists act as advice-givers and recommend the diet a patient should follow. While both stable and improved patients have HbA1c levels below 7% at the conclusion of the period of scope of this study, appointments with nutritionists appeared to be effective in ensuring patients remain stable.

In both specific and grouped referrals, highly decompensated patients showed lower adherence than the rest of the segments. In particular, the doctor–nurse referral of highly decompensated patients showed adherence levels statistically lower than all others, including moderately decompensated patients. The difference in the severity of decompensation among highly decompensated patients could be explained by the lack of timely appointments with nurses. To treat T2DM, the role of nurses consisted of conducting regular tests on patients and, in certain circumstances, undertaking treatment sessions for diabetic foot. The non-attendance of these appointments can be detrimental [31], while the non-treatment of diabetic foot can lead to severe complications [32].

Results reveal there was a relationship between the evolution segments and compliance with scheduled appointments. Improved patients were more compliant with referrals made by doctors or nurses, whereas stable patients were more compliant when they were referred by nutritionists. Similarly, improved patients were more compliant with their next check-up when it was with a doctor or nurse, whereas stable patients were more compliant when they had their next check-up with a nutritionist. On the other hand, highly decompensated patients were less compliant with regard to all referrals. This illustrated that failing to attend check-ups in a timely manner caused patients to become decompensated, as outlined in Reference [18].

While results showed a relationship between the evolution segments and adherence, it could not be established for certain whether this relationship was causal. This study is important because it allows healthcare professionals to observe patient compliance. In turn, this means they can focus particular attention on patients who are failing to attend their appointments in a timely manner, since it is precisely these individuals who may be experiencing health-related complications. In addition, encouraging greater compliance with appointments results in fewer wasted resources. For example, it prevents professionals wasting their time having to search for patient medical records, and it ensures professionals use their time more productively [33].

The main limitation of this study relates to the quality of the data provided by the respective information systems. While their databases store data about appointments, they do not explain the true nature of events in great detail. All appointments considered in this study were explicitly scheduled and marked as cardiovascular check-ups in the information systems. However, this did not ensure that all check-ups have been recorded, since patients could have been seen via a non-scheduled appointment or during an appointment with a specialist beyond the treatment indicated by the health center. On the other hand, the appointments attended by patients were not necessarily due to referrals made by the healthcare team. It was possible that a patient had visited the health center for another reason and decided to have a cardiovascular check-up at the same time. The availability of professionals at the time of taking an appointment could cause a bias in the sample. A patient who must reschedule an appointment with a professional that did not have available hours would probably be considered as a non-adherent patient in the study. Another aspect not studied was how severity or comorbidity affected the adherence, e.g., whether a greater severity implied a greater adherence of the patient. A further limitation related to additional factors not considered in the case study. For example, several of the results outlined could be affected by external factors or the individual characteristics of patients, such as food habits, quality of life, metabolism, availability of healthcare professionals, medication consumed and adherence thereto, among others. Finally, the number of interviewed patients was small, and there may have been a bias towards patients with higher adherence, since the patients were interviewed at the healthcare centers.

## 5. Conclusions

Undertaking this case study has helped to identify a relationship between the evolution of patients with adherence to treatment at the level of medical referrals. Patients who attended their medical appointments in a timely manner tended to improve and become stable, while those who failed to attend often became decompensated. Stable patients tended to comply more frequently with appointments with nutritionists compared to improved patients. Highly decompensated patients showed the lowest adherence to referrals, whether grouped or specific, compared to the other segments. Regardless of which discipline made the referral, highly decompensated patients failed to attend appointments in a timely manner. In general terms, patients were more adherent when they were referred to a doctor, although when a doctor referred them to another professional discipline, they became less adherent. The results outlined did not provide evidence of causality, but they did show a correlation between timely attendance of appointments and evolution.

This study provided an adherence analysis by considering the transitions of a patient between different healthcare disciplines, and therefore this approach contained a more detailed analysis of the discipline to which a patient was most adherent. Future work could replicate this study in other healthcare centers with other patients in order to confirm whether the trend continues. This would ensure that the problem of diabetes is addressed from other perspectives, while promoting public policy and redesigning protocols to ensure the ongoing improvement of people living with this disease.

## Figures and Tables

**Table 1:** Adherence to grouped referrals from one particular discipline to any other discipline.

Metric	Referral	# Referrals	Adherence
$F_{\mathrm{doctor}}$	Doctor–Any	14,237	62.60% ^1^
$F_{\mathrm{nurse}}$	Nurse–Any	6807	65.04%
$F_{\mathrm{nutritionist}}$	Nutritionist–Any	2717	68.46% ^1^
C	Overall population	23,761	63.97%

^1^ Values are statistically significant to an accuracy of 95%.

**Table 2:** Adherence to grouped referrals from any discipline to one discipline in particular.

Metric	Referral	# Referrals	Adherence
$T_{\mathrm{doctor}}$	Any–Doctor	13,929	68.23% ^1^
$T_{\mathrm{nurse}}$	Any–Nurse	7471	57.88% ^1^
$T_{\mathrm{nutritionist}}$	Any–Nutritionist	2361	58.14% ^1^
C	Overall population	23,761	63.97%

^1^ Values are statistically significant to an accuracy of 95%

**Table 3:** Specific referral adherence.

Metric	Referral	# Referrals	Adherence
$S_{\mathrm{doctor},\mathrm{doctor}}$	Doctor–Doctor	7304	68.00% ^1^
$S_{\mathrm{doctor},\mathrm{nurse}}$	Doctor–Nurse	5462	57.09% ^1^
$S_{\mathrm{doctor},\mathrm{nutritionist}}$	Doctor–Nutritionist	1471	56.29% ^1^
$S_{\mathrm{nurse},\mathrm{doctor}}$	Nurse–Doctor	5039	66.96% ^1^
$S_{\mathrm{nurse},\mathrm{nurse}}$	Nurse–Nurse	1098	56.83% ^1^
$S_{\mathrm{nurse},\mathrm{nutritionist}}$	Nurse–Nutritionist	670	64.03%
$S_{\mathrm{nutritionist},\mathrm{doctor}}$	Nutritionist–Doctor	1586	73.33% ^1^
$S_{\mathrm{nutritionist},\mathrm{nurse}}$	Nutritionist–Nurse	911	63.89%
$S_{\mathrm{nutritionist},\mathrm{nutritionist}}$	Nutritionist–Nutritionist	220	52.27% ^1^
C	Overall population	23,761	63.97%

^1^ Values are statistically significant to an accuracy of 95%.

**Table 4:** Evolution segment adherence for group referrals from a specific discipline to any other discipline.

Segment	$F_{doctor}$	$F_{nurse}$	$F_{nutritionist}$	# Referrals	General Adherence
Highly Decompensated	61.12% ^1^	62.84% ^1^	64.83% ^1^	10,924	61.99% ^1^
Moderately Decompensated	62.91%	66.04%	70.52% ^2^	3527	64.64%
Stable	63.30% ^2^	66.23%	72.93% ^1,2^	5983	65.35% ^1^
Improved	66.21% ^1^	68.49% ^1^	69.39%	3327	67.27% ^1^
Overall Population	62.60%	65.04%	68.46%	23,761	63.97%

^1^ Values are statistically significant to an accuracy of 95%, regarding the adherence of the overall population. ^2^ values are statistically significant to an accuracy of 95%, regarding the general adherence of the segment.

**Table 5:** Evolution segment adherence for grouped referrals from any discipline to a specific discipline.

Segment	$T_{doctor}$	$T_{nurse}$	$T_{nutritionist}$	# Referrals	General Adherence
Highly Decompensated	66.83% ^1,2^	53.85% ^1,2^	54.46% ^2^	10,924	61.99%
Moderately Decompensated	68.60% ^2^	59.48% ^2^	58.56% ^2^	3527	64.64%
Stable	68.62% ^2^	61.27% ^1,2^	62.60% ^1^	5983	65.35%
Improved	72.23% ^1,2^	61.33% ^1,2^	59.44% ^2^	3327	67.27%
Overall Population	68.23%	57.88%	58.14%	23,761	63.97%

^1^ Values are statistically significant to an accuracy of 95%, regarding the adherence of the overall population. ^2^ values are statistically significant to an accuracy of 95%, regarding the general adherence of the segment.

## References

[1] International Diabetes Federation (2015). IDF Diabetes Atlas. https://www.idf.org/e-library/epidemiology-research/diabetes-atlas/13-diabetes-atlas-seventh-edition.html.

[2] Ministerio de Salud de Chile Guía Clínica: Diabetes Mellitus Tipo 2. Chile, 2010. http://www.minsal.cl/portal/url/item/72213ed52c3e23d1e04001011f011398.pdf.

[3] Ko S.H., Park S., Cho J.H., Ko S.H., Shin K.M., Lee S.H., Song K.H., Park Y.M., Ahn Y.B. (2012). Influence of the Duration of Diabetes on the Outcome of a Diabetes Self-Management Education Program. Diabetes Metab. J..

[4] Williams G.C., Zeldman A. (2002). Patient-centered diabetes self-management education. Curr. Diabetes Rep..

[5] Sabaté E. (2003). Adherence to Long-Term Therapies: Evidence for Action.

[6] Vermeire E., Hearnshaw H., Van Royen P., Denekens J. (2001). Patient adherence to treatment: Three decades of research. A comprehensive review. J. Clin. Pharm. Ther..

[7] Gillett M., Dallosso H.M., Dixon S., Brennan A., Carey M.E., Campbell M.J., Heller S., Khunti K., Skinner T.C., Davies M.J. (2010). Delivering the diabetes education and self management for ongoing and newly diagnosed (DESMOND) programme for people with newly diagnosed type 2 diabetes: Cost effectiveness analysis. Br. Med. J..

[8] De León A., Castillo Rodríguez J.C., del Domínguez Coello S., Pérez R., del Cristo M., Brito Díaz B., Borges Álamo C., Carrillo Fernández L., Almeida González D., Alemán Sánchez J.J. (2009). Estilo de vida y adherencia al tratamiento de la población canaria con diabetes mellitus tipo 2. Rev. Española Salud Pública.

[9] Toth E.L., Majumdar S.R., Guirguis L.M., Lewanczuk R.Z., Lee T.K., Johnson J.A. (2003). Compliance with clinical practice guidelines for type 2 diabetes in rural patients: Treatment gaps and opportunities for improvement. Pharmacotherapy.

[10] Hoyos T.N., Arteaga M.V., Muñoz M. (2011). Factores de no adherencia al tratamiento en personas con Diabetes Mellitus tipo 2 en el domicilio. La visión del cuidador familiar. Investig. Educ. Enfermería.

[11] Khowaja M.A. (2012). Treatment Compliance to Diabetes Care: A Cross-sectional Study. Can. J. Diabetes.

[12] Parris E.S., Lawrence D.B., Mohn L.A., Long L.B. (2005). Adherence to Statin Therapy and LDL Cholesterol Goal Attainment by Patients With Diabetes and Dyslipidemia. Diabetes Care.

[13] Efron B., Feldman D. (1991). Compliance as an explanatory variable in clinical trials. J. Am. Stat. Assoc..

[14] Horwitz R.I., Viscoli C.M., Donaldson R.M., Murray C.J., Ransohoff D.F., Berkman L., Horwitz S.M., Sindelar J. (1990). Treatment adherence and risk of death after a myocardial infarction. Lancet.

[15] Donnan P.T., MacDonald T.M., Morris A.D. (2002). Adherence to prescribed oral hypoglycaemic medication in a population of patients with Type 2 diabetes: A retrospective cohort study. Diabet. Med..

[16] Dailey G., Kim M.S., Lian J.F. (2001). Patient compliance and persistence with antihyperglycemic drug regimens: Evaluation of a medicaid patient population with type 2 diabetes mellitus. Clin. Ther..

[17] Ciechanowski P.S., Katon W.J., Russo J.E., Walker E.A. (2001). The patient-provider relationship: Attachment theory and adherence to treatment in diabetes. Am. J. Psychiatry.

[18] Schectman J.M., Schorling J.B., Voss J.D. (2008). Appointment adherence and disparities in outcomes among patients with diabetes. J. Gen. Intern. Med..

[19] Norell S.E. (1981). Accuracy of patient interviews and estimates by clinical staff in determining medication compliance. Soc. Sci. Med. Part E Med. Psychol..

[20] DiMatteo M.R., DiNicola D.D. (1983). Achieving patient compliance: The psychology of the medical practitioner’s role. JAMA.

[21] Roth H.P., Caron H.S. (1978). Accuracy of doctors’ estimates and patients’ statements on adherence to a drug regimen. Clin. Pharmacol. Ther..

[22] Morisky D.E., Green L.W., Levine D.M. (1986). Concurrent and predictive validity of a self-reported measure of medication adherence. Med. Care.

[23] Farmer K.C. (1999). Methods for measuring and monitoring medication regimen adherence in clinical trials and clinical practice. Clin. Ther..

[24] Phillips J.D. (2008). Evaluating Patient Compliance: Effect of Appointment Reminder Systems on Attendance.

[25] Lange I., Campos S., Urrutia M., Bustamante C., Alcayaga C., Tellez Á., PÉREZ J.C., Villarroel L., Chamorro G., Piette J. (2010). Efecto de un modelo de apoyo telefónico en el auto-manejo y control metabólico de la Diabetes tipo 2, en un Centro de Atención Primaria, Santiago, Chile. Rev. Méd. Chile.

[26] Conca T., Saint-Pierre C., Herskovic V., Sepúlveda M., Capurro D., Prieto F., Fernandez-Llatas C. (2018). Multidisciplinary Collaboration in the Treatment of Patients With Type 2 Diabetes in Primary Care: Analysis Using Process Mining. J. Med. Internet Res..

[27] Van der Aalst W.M.P. (2016). Process Mining: Data Science in Action.

[28] Melnikow J., Kiefe C. (1994). Patient compliance and medical research. J. Gen. Intern. Med..

[29] Braun V., Clarke V. (2006). Using thematic analysis in psychology. Qual. Res. Psychol..

[30] Wagner E.H., Sandhu N., Newton K.M., McCulloch D.K., Ramsey S.D., Grothaus L.C. (2001). Effect of improved glycemic control on health care costs and utilization. JAMA.

[31] American Diabetes Association (2014). Standards of medical care in diabetes—2014. Diabetes Care.

[32] Boulton A.J.M., Vileikyte L., Ragnarson-Tennvall G., Apelqvist J. (2005). The global burden of diabetic foot disease. Lancet.

[33] Grover S., Gagnon G., Flegel K.M., Hoey J.R. (1983). Improving appointment-keeping by patients new to a hospital medical clinic with telephone or mailed reminders. Can. Med. Assoc. J..

